# A 0.82 μVrms ultralow 1/*f* noise bandgap reference for a MEMS gyroscope

**DOI:** 10.1038/s41378-023-00505-3

**Published:** 2023-04-17

**Authors:** Junjun Zou, Qi Wei, Chunge Ju, Hua Liao, Haoyu Gu, Bowen Xing, Bin Zhou, Rong Zhang

**Affiliations:** Department of Precision Instruments, Tsinghua University, Beijing, 100084 China

**Keywords:** Electrical and electronic engineering, Nanoscience and technology

## Abstract

High-precision microelectromechanical system (MEMS) gyroscopes are significant in many applications. Bias instability (BI) is an important parameter that indicates the performance of a MEMS gyroscope and is affected by the 1/*f* noise of the MEMS resonator and readout circuit. Since the bandgap reference (BGR) is an important block in the readout circuit, reducing its 1/*f* noise is key to improving a gyroscope’s BI. In a traditional BGR, the error amplifier is applied to provide a virtual short-circuit point, but it introduces the main low-frequency noise sources. This paper proposes an ultralow 1/*f* noise BGR by removing the error amplifier and applying an optimized circuit topology. In addition, a simplified but accurate noise model of the proposed BGR is obtained to optimize the BGR’s output noise performance. To verify this design, the proposed BGR has been implemented in a 180 nm CMOS process with a chip area of 545 × 423 μm. The experimental results show that the BGR’s output integrated noise from 0.1 to 10 Hz is 0.82 μV and the thermal noise is 35 nV/√Hz. Furthermore, bias stability tests of the MEMS gyroscope fabricated in our laboratory with the proposed BGR and some commercial BGRs are carried out. Statistical results show that reducing the BGR’s 1/f noise can nearly linearly improve the gyroscope’s BI.

## Introduction

With the advantages of small size and high precision, high-performance MEMS gyroscopes are increasingly being applied in many applications, including inertial navigation, automobiles, and smartphones. In navigation applications, Bias instability (BI), the lowest bias error calculated by the Allan curve^1^, is a critical parameter used to indicate the performance of a MEMS gyroscope. The MEMS gyroscope’s BI is mainly determined by the 1/*f* noise of the MEMS resonator and readout circuit. With the development of MEMS resonators, readout circuit noise has become the bottleneck to improving BI^2–5^. Since the BGR is an important block in the readout circuit for providing a reference voltage, its 1/*f* noise is a key factor affecting the BI. Therefore, a low 1/*f* noise BGR is an attractive proposition for achieving a high-performance MEMS gyroscope.

**Fig. 1:**
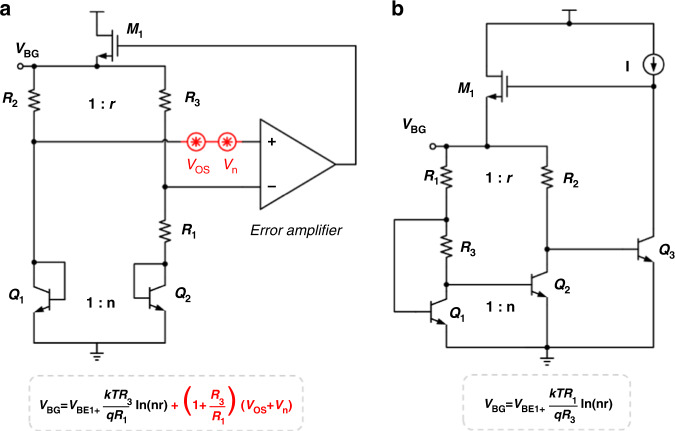
Comparison of traditional BGR and proposed BGR. **a** Traditional voltage-mode BGR circuit. **b** Simplified topology of the proposed BGR

Voltage-mode BGRs and current-mode BGRs are the two main kinds of BGRs. In current-mode BGRs, a current mirror is used to produce a current nearly invariant to temperature. However, the device mismatch and 1/*f* noise of the current mirror increase the BGR’s output noise. Therefore, compared with current-mode BGRs, voltage-mode BGRs are better candidates to achieve low noise^6,7^. A traditional voltage-mode BGR circuit is illustrated in Fig. 1a^8^. The error amplifier is applied to provide a virtual short-circuit point, but its 1/*f* noise is the main low-frequency noise source since it is multiplied by a large amplifying factor 1 + *R*_*3*_/*R*_*1*_ (usually more than 10). In previous studies, some methods have been proposed to reduce noise. A chopping technique with low-pass filters was applied in refs. ^9,10^, which modulates low-frequency noise to the high-frequency region and subsequently filters the noise using low-pass filters. However, modulators and filters increase power consumption and circuit complexity. An autozeroing technique^11,12^ was proposed to remove the offset and noise of the error amplifier. However, this is not appropriate for continuous-time applications. Lianxi Liu et al.^13^ proposed a noise suppression technique to reduce the amplifying factor. However, the factor is still greater than 1, and it cannot eliminate the offset and noise completely. Analog devices^14^ proposed a low-noise BGR topology based on dual-threshold junction field-effect transistor (JFET) technology. Since JFETs exhibit a better low-frequency noise performance than complementary metal-oxide-semiconductor field-effect transistors (CMOSFETs), BGRs based on JFETs can achieve low 1/*f* noise but require a special process.

This paper proposes an ultralow 1/*f* noise BGR for improving gyroscope performance. The simplified topology is illustrated in Fig. 1b. Compared with a traditional voltage-mode BGR, it removes the error amplifier to reduce the offset and 1/*f* noise. In addition, to further optimize the output noise performance, we present a simplified but accurate noise model of the proposed BGR and optimize the device parameters to suppress output noise. The noise measurement results verify the low 1/*f* noise characteristics of the proposed BGR. Furthermore, to verify the BGR’s effect on the gyroscope’s BI, with the proposed BGR and commercial BGRs, which have different output 1/*f* noise, a bias stability test of the MEMS gyroscope fabricated in our laboratory is carried out. The experimental results demonstrate the effect of reducing the BGR’s 1/*f* noise on improving the gyroscope’s BI.

## Results

### MEMS gyroscope

**Fig. 2:**
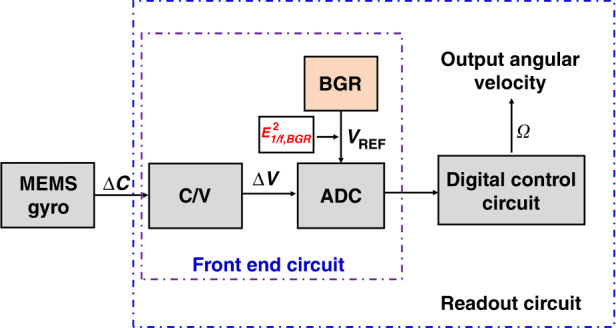
The block diagram of the MEMS gyroscope

The block diagram of the MEMS gyroscope is illustrated in Fig. 2. With an angular velocity input, the MEMS gyroscope produces a capacitance variation *∆C* due to the Coriolis force, and *∆C* is proportional to the angular velocity. When *∆C* is digitalized by the front-end circuit, the digital control circuit demodulates and compensates the digital information *D*_*n*_ and obtains the output angular velocity *Ω*.

The noise of readout circuits directly affects MEMS gyroscope performance, and the bias instability of MEMS gyroscopes is mainly contributed by the flicker noise in the readout circuit^2^. Since the BGR provides a reference voltage for the analog-to-digital converter in the front-end circuit, BGR flicker noise is a key factor affecting the bias instability of the MEMS gyroscope.

### Basic principle of the proposed BGR

The BGR operates based on the characteristics of bipolar transistors. When bipolar transistors operate in the forward active region^15^, the base-emitter voltage *V*_BE_ can be described as a function of the collector current IC and absolute temperature *T*.

1 $$V_{{\mathrm{BE}}} = V_{g0}\left( {1 - \frac{T}{{T_0}}} \right) + V_{{\mathrm{BE}}0}\left( {\frac{T}{{T_0}}} \right) - n\frac{{kT}}{q}\ln \left( {\frac{T}{{T_0}}} \right) + \frac{{kT}}{q}\ln \left( {\frac{{I_c}}{{I_{{{{\mathrm{c}}}}0}}}} \right)$$

where *V*_*g0*_ is the extrapolated energy bandgap voltage at absolute zero, *k* is the Boltzmann constant, *q* is the electron charge, n is the constant process parameter, and *V*_BE0_ is the base-emitter voltage at *T*_*0*_ and *I*_*c0*_. Since *V*_*g0*_ > *V*_*BE0*_ + *kT*_*0*_/*q* × ln(*I*_*c*_/*I*_*c0*_), *V*_BE_ can be nearly considered a complementary-to-absolute temperature term. The base-emitter voltage differential *ΔV*_BE_ of two transistors, which have different emitter cross-sectional areas and operate at different current densities, can be expressed as (2).

2 $$\Delta V_{{\mathrm{BE}}} = \frac{{kT}}{q}\ln \left( {\frac{{I_{c1}A_2}}{{I_{c2}A_1}}} \right)$$

where *A*_*1*_ and *A*_*2*_ are the emitter cross-sectional areas of the two transistors, and *I*_*c1*_ and *I*_*c2*_ are the collector currents of the two transistors. Therefore, *V*_BE_ plus a voltage proportional to *ΔV*_BE_ can eliminate the first-order temperature term and form a reference voltage approximately equal to *V*_*g0*_, which is the basic principle of the BGR. In Fig. 1a, for traditional voltage-mode BGRs, an error amplifier composed of MOS transistors is applied to form a base-emitter voltage differential on *R*_*1*_. Furthermore, the amplifier can help traditional voltage-mode BGRs achieve a good power supply rejection ratio (PSRR) performance since it provides a negative feedback loop to reduce the effect of other signals on the output reference voltage. However, due to device mismatches and process variation, the error amplifier introduces input offset and 1/*f* noise, which is then amplified by a large factor (1 + *R*_*3*_*/R*_*1*_). Therefore, traditional voltage-mode BGRs are not appropriate for ultralow noise applications.

**Fig. 3:**
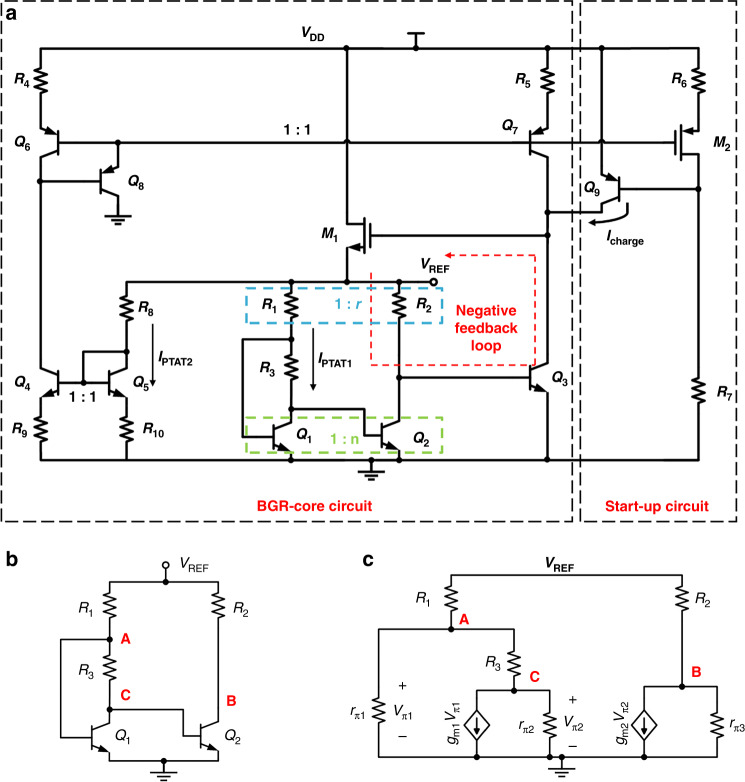
Specific topology of proposed BGR. **a** Schematic of the proposed BGR. **b** Simplified circuit of the proposed BGR. **c** Corresponded small-signal model of the simplified circuit

The specific schematic of the proposed BGR^16^ is illustrated in Fig. 3a. This design avoids applying an error amplifier to form a *ΔV*_BE_. In the BGR-core circuit, transistors *Q*_*1*_ and *Q*_*2*_ form a *ΔV*_BE_ on *R*_*3*_. Neglecting the base current of bipolar transistors, the voltage on *R*_*1*_ is *R*_*1*_*/R*_*3*_ × *ΔV*_BE_. Therefore, the output reference voltage *V*_REF_ is a *V*_BE_ plus a voltage proportional to *ΔV*_BE_, and it can be expressed as (3).

3 $$V_{{\mathrm{REF}}} = V_{{\mathrm{BE}}1} + \frac{{k{\mathrm{TR}}_1}}{{qR_3}}\ln \left( {\frac{{I_{c1}A_2}}{{I_{c2}A_1}}} \right) = V_{{\mathrm{BE}}1} + \frac{{k{\mathrm{TR}}_1}}{{qR_3}}\ln \left( {nr\frac{{V_{c1}}}{{V_{c2}}}} \right)$$

where *n* is the emitter cross-sectional area ratio of *Q*_*2*_ versus *Q*_*1*_, *r* is the resistance ratio of *R*_*2*_ versus *R*_*1*_, *V*_*c1*_ and *V*_*c2*_ are the voltages on *R*_*1*_ and *R*_*2*_, *A*_*1*_ and *A*_*2*_ are the emitter cross-sectional areas of *Q*_*1*_ and *Q*_*2*_, and *I*_*c1*_ and *I*_*c2*_ are the collector currents of *Q*_*1*_ and *Q*_*2*_. By adjusting *n*, *r*, and the ratio of *R*_*1*_ and *R*_*3*_, the output voltage can be approximately equal to *V*_*g0*_.

Resistors *R*_*1*_, *R*_*2*_, and *R*_*3*_ and transistors *Q*_*1*_, *Q*_*2*_, and *Q*_*3*_ form a negative feedback loop. The loop is applied to reduce the effect of other signal sources on the output voltage. In addition, the branch of *Q*_*5*_, *R*_*8*_ and *R*_*10*_ is utilized to produce another current *I*_PTAT2_ proportional to absolute temperature (PTAT). Then, *I*_PTAT2_ is duplicated to transistor *Q*_*3*_ by the current mirrors formed by transistors *Q*_*4*_ and *Q*_*5*_ and transistors *Q*_*6*_ and *Q*_*7*_. Consequently, the collector current of *Q*_*3*_ is directly affected by *I*_PTAT2_ rather than the supply voltage. Therefore, without an error amplifier, the negative feedback loop and the PTAT current *I*_PTAT2_ can help the BGR achieve good PSRR performance. The proposed BGR can remove the input offset and 1/*f* noise introduced by the error amplifier.

Due to the negative feedback loop and the source follower *M*_*1*_, the proposed BGR has characteristics of low output resistance and strong driving ability. A start-up circuit is applied to help the BGR work in a normal state. When the BGR is powered on, a current *I*_charge_ produced by transistor *Q*_*9*_ is injected into the BGR-core circuit. As transistors *Q*_*6*_, *Q*_*7*_, and *M*_*2*_ turn on and the BGR works normally, the base voltage of *Q*_*9*_ increases to a large value, and *Q*_*9* finally_ turns off.

### 1/*f* Noise suppression

Although an error amplifier is not applied in the design, the two current mirrors can also introduce low-frequency noise at node B in Fig. 3a. However, the 1/*f* noise introduced by bipolar transistors is much lower than that introduced by CMOS transistors. Furthermore, the topology can achieve an amplification factor of less than 1 to suppress the 1/*f* noise.

To analyze the amplification factor, a simplified circuit of the proposed BGR is illustrated in Fig. 3b, and the corresponding small-signal model is shown in Fig. 3c.

The amplification factor *α* is defined as the gain of the 1/*f* noise *V*_*n,B*_ at node B transferred to the output node. It can be expressed as (4).

4 $$\alpha = \frac{{V_{n,{\mathrm{REF}}}}}{{V_{n,B}}}$$

According to Kirchhoff’s law, the 1/*f* noise at nodes A and C and the output reference transferred from node B can be expressed as (5), (6), and (7).

5 $$V_{n,C} = \frac{{\left( {1 - g_{m1}R_3} \right)r_{\pi 2}}}{{R_3 + r_{\pi 2}}}V_{n,A}$$

6 $$V_{n,A} = \frac{{\left( {R_3 + r_{\pi 2}} \right)r_{\pi 1} \times V_{n,REF}}}{{\left( {R_1 + r_{\pi 1}} \right)\left( {R_3 + r_{\pi 2}} \right) + R_1r_{\pi 1}\left( {1 + g_{m1}r_{\pi 2}} \right)}}$$

7 $$V_{n,{\mathrm{REF}}} = g_{m2}R_2V_{n,C} + \left( {1 + \frac{{R_2}}{{r_{\pi 3}}}} \right)V_{n,B}$$

where *g*_*m1*_ and *g*_*m2*_ are the transconductances of *Q*_*1*_ and *Q*_*2*_, and *r*_*π1*_, *r*_*π2*_, and *r*_*π3*_ are the input resistors of *Q*_*1*_, *Q*_*2*_ and *Q*_*3*_. Substituting (5), (6), (7) into (4), the amplification can be expressed as (8).

8 $$\alpha = \left( {\frac{{r_{\pi 3} + R_2}}{{r_{\pi 3}}}} \right) \times \frac{{\left( {r_{\pi 3} + R_1} \right)\left( {r_{\pi 2} + R_3} \right) + R_1r_{\pi 1}\left( {1 + g_{m1}r_{\pi 2}} \right)}}{{g_{m2}R_2r_{\pi 1}r_{\pi 2}\left( {g_{m1}R_3 - 1} \right) + \left( {r_{\pi 1} + R_1} \right)\left( {r_{\pi 2} + R_3} \right) + R_1r_{\pi 1}\left( {1 + g_{m1}r_{\pi 2}} \right)}}$$

According to the characteristics of bipolar transistors presented in ref. ^17^,

9 $$g_{m,i} = \frac{{qI_{c,i}}}{{kT}},i = 1,2,3.$$

10 $$r_{\pi ,i} = \frac{{\beta _i}}{{g_{m,i}}},i = 1,2,3.$$

Substituting (9), (10) into (8), *α* can be expressed as (11).

11 $$\alpha \approx \left( {\frac{{R_2 + r_{\pi 3}}}{{r_{\pi 3}}}} \right) \times \frac{1}{{\ln \left( {\frac{{V_{c1}}}{{V_{c2}}}nr} \right) - 1}}$$

Therefore, *α* can be reduced by increasing *r*_*π3*_, *n*, and *r*. According to (9), (10), *r*_*π3*_ is inversely proportional to the collector current of *Q*_*3*_. When *r*_*π3*_ is increased to a comparative resistor by reducing *I*_PTAT2_, (*R*_*2*_ + *r*_*π3*_)/*r*_*π3*_ is nearly equal to 1. In addition, since *V*_*c1*_ roughly equals *V*_*c2*_, large *n* and *r* can be applied to reduce *α* and ensure that *α* is much less than 1. Consequently, the design can apply a small current *I*_PTAT2_ and large *n* and *r* to effectively suppress the 1/*f* noise introduced by the two current mirrors.

### Simplified but accurate output noise model

**Fig. 4:**
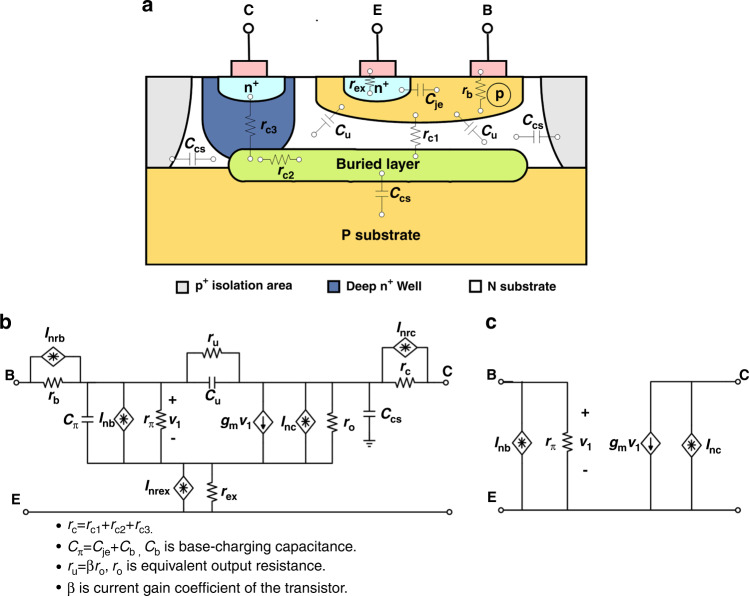
Noise model of typical *npn* bipolar transistor. **a** A cross-section of a typical *npn* bipolar transistor with parasitic resistors and capacitors. **b** Corresponding equivalent noise model. **c** Simplified noise model

A cross-section of a typical *npn* bipolar transistor with parasitic resistors and capacitors is illustrated in Fig. 4a. Figure 4b presents the corresponding equivalent noise model. Since the low-frequency region is our focus and the noise model in Fig. 4b is quite complicated, the parasitic capacitors and small parasitic resistors are removed. The simplified noise model of the *npn* bipolar transistor is shown in Fig. 4c.

**Fig. 5:**
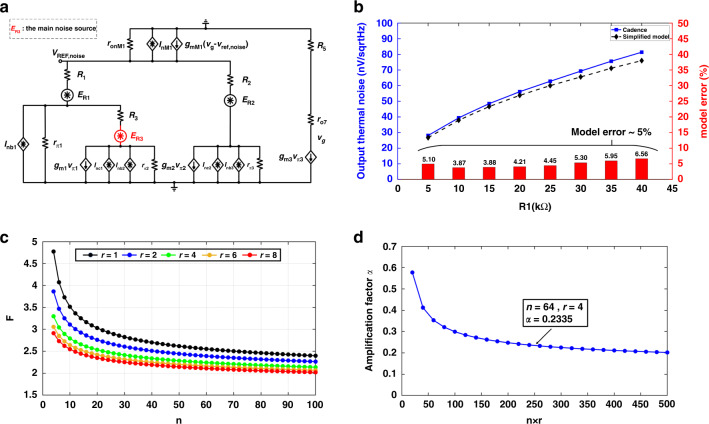
Noise optimization. **a** The small-signal model of the proposed BGR with RMS noise sources. **b** The simulated output noise calculated by Cadence and the simplified model. **c** The relationship between *F* and *n*, *r*. **d** The relationship between the amplification factor *α* and *n* × *r*

To further optimize the noise performance of this design, the effect of individual noise sources on output is analyzed. Since the noise of the two current mirrors is effectively suppressed, it is not included in the analysis. The small-signal model with RMS noise sources is presented in Fig. 5a to calculate the output noise of the proposed BGR. According to the theory proposed by ref. ^18^, the spectral density of individual noise sources in Fig. 5a is

12 $$E_{R,i} = \sqrt {4kTR_i + \frac{{K_1}}{f}\left( {I_{c,i}R_i} \right)^2} ,i = 1,2,3.$$

13 $$I_{{\mathrm{nb}},i} = \sqrt {2q \times \frac{{I_{c,i}}}{{\beta _i}} + \frac{{K_2}}{f}\left( {\frac{{I_{c,i}}}{{\beta _i}}} \right)^{\alpha _1}} ,i = 1,2,3.$$

14 $$I_{{\mathrm{nc}},i} = \sqrt {2qI_{c,i}} ,i = 1,2.$$

15 $$I_{n,M_1} = \sqrt {4kT \times \left( {\frac{2}{3}g_{m,M_1}} \right) + \frac{{K_3}}{f} \times I_D^{\alpha _2}}$$

where *K*_*1*_, *K*_*2*_, and *K*_*3*_ are process parameters inversely proportional to the device area, *α*_*1*_ and *α*_*2*_ are process constants in the range 0.5 to 2, *I*_nc*,i*_ is the current noise due to the collector current, *I*_nb*,i*_ is the current noise due to the base current and process, *I*_*n,M1*_ is the current noise due to the drain current and process, *β*_*i*_ is the current gain factor of transistor *Q*_*i*_, and *g*_*m,M1*_ is the transconductance of *M*_*1*_. Since every single noise source is uncorrelated, the contribution of all noise sources can be superposed directly to calculate the output reference noise.

According to Kirchhoff’s law, the total RMS output reference noise *V*_REFnoise_ is as shown in (16).

16 $$V_{{\mathrm{REFnoise}}} = \sqrt{\begin{array}{l}\left( {\frac{{X_1}}{{X_D}}} \right)^2 \times \left( {I_{{\mathrm{nb}}2}^2R_l^2 + I_{{\mathrm{nc}}1}^2R_1^2} \right) + \left( {\frac{{X_2}}{{X_D}}} \right)^2 \times \left( {I_{{\mathrm{nb}}1}^2R_1^2 + E_{R1}^2} \right) + \left( {\frac{{X_3}}{{X_D}}} \right)^2 \times S_{R3}^2\\ \qquad\qquad\qquad\qquad + \left( {\frac{{X_4}}{{X_D}}} \right)^2 \times \left( {E_{R2}^2 + I_{{\mathrm{nc}}2}^2R_2^2 + \left( {\frac{{T_{nM1}R_1}}{{X_5}}} \right)^2} \right)\end{array}}$$

The *X* factors in (16) are

17 $$X_1 = g_{m2}R_2R_3^2\left( {r_{\pi 2}{{{\mathrm{||}}}}R_3} \right)$$

18 $$X_2 = g_{m2}R_2R_3\left( {r_{\pi 2}\left\| {R_3} \right.} \right)\left( {R_1\left\| {r_{\pi 1}} \right\|R_3} \right)\left( {g_{m1}R_3 - 1} \right)$$

19 $$X_3 = \frac{{R_1}}{{R_3}}\left( {X_1 + X_2} \right)$$

20 $$X_4 = R_1R_3^2 + R_1\left( {r_{\pi 2}{{{\mathrm{||}}}}R_3} \right)\left( {R_1{{{\mathrm{||}}}}r_{\pi 1}{{{\mathrm{||}}}}R_3} \right)\left( {g_{m1}R_3 - 1} \right)$$

21 $$X_{{{\mathrm{5}}}} = g_{m1}\left( {r_{\pi 3}||R_2} \right)g_{m3}\left( {r_{o7} + R_5} \right)$$

22 $$X_D = X_2 + X_4$$

where *r*_*o7*_ is the equivalent output resistor of *Q*_*7*_. Since the noise models of bipolar transistors are simplified to obtain the output noise model of the proposed BGR, to verify the accuracy of the noise model, output noise is calculated by Cadence and the expression in (17). The calculation of Cadence uses complex and accurate models of bipolar transistors. The simulation results are illustrated in Fig. 5b. Under different resistances of *R*_*1*_, the error of the simplified model is approximately 5%. Therefore, the simplified model is accurate and can be applied to analyze the noise performance of the proposed BGR.

### Noise optimization

In the low-frequency region, 1/*f* noise is much more significant than thermal noise. Therefore, the thermal noise caused by resistors and bipolar transistors is not the main focus. In addition, since *r*_*o7*_ has a large resistance, *X*_*5*_ is much greater than 1, and the 1/*f* noise caused by *M*_*1*_ can be neglected.

According to (17)–(20), *X*_*3*_ > *X*_*4*_ > *X*_*2*_ > *X*_*1*_. Therefore, the noise caused by *R*_*3*_ needs to be the main focus. The 1/*f* noise caused by *R*_*3*_ to the output node can be expressed as (23).

23 $$\begin{array}{c}\frac{{X_3}}{{X_D}} \times \sqrt {\frac{{K_1}}{f}\left( {I_{c1}R_3} \right)^2} = \frac{{r\beta _2 \times \sqrt {\frac{{V_{c1}}}{{V_T}}\ln \left( {\frac{{V_{c1}}}{{V_{c2}}}nr} \right)} }}{{\ln \left( {\frac{{V_{c1}}}{{V_{c2}}}nr} \right)\left( {r\beta _2 + 1} \right) + r\beta _2\left( {\frac{{V_{c1}}}{{V_{c2}}} - 1} \right)}} \times \sqrt {\frac{K}{f}} V_{c1}\\ = F\left( {n,r} \right) \times \sqrt {\frac{K}{f}} V_{c1}\end{array}$$

where *F* is a factor that varies with *n* and *r* and *K* is a constant process parameter. Based on (24), the effect of noise by *R*_*3*_ on the output node is related to the factors *n* and *r*. Figure 5c shows the amplitude of *F* under different *n* and *r*. Large *n* and *r* can effectively reduce *F* and thus reduce the 1/*f* noise of the proposed BGR. Figure 5d shows the relationship between the amplification factor *α* and *n* × *r*, and large *n* and *r* can also effectively reduce *α*. Since *n* is the emitter cross-sectional area ratio of *Q*_*2*_ versus *Q*_*1*_, a large *n* means consuming a large chip area. A comparatively large *r* can cause a mismatch between *R*_*1*_ and *R*_*2*_ and thus introduce a high-order temperature term to deteriorate TC. Therefore, this design chooses *n* = 64 and *r* = 4 to obtain a low amplification factor *α* and optimize the output noise performance. Furthermore, polysilicon resistors have been chosen to implement this BGR due to the low process parameter *K*^19^.

### Proposed BGR performance

**Fig. 6:**
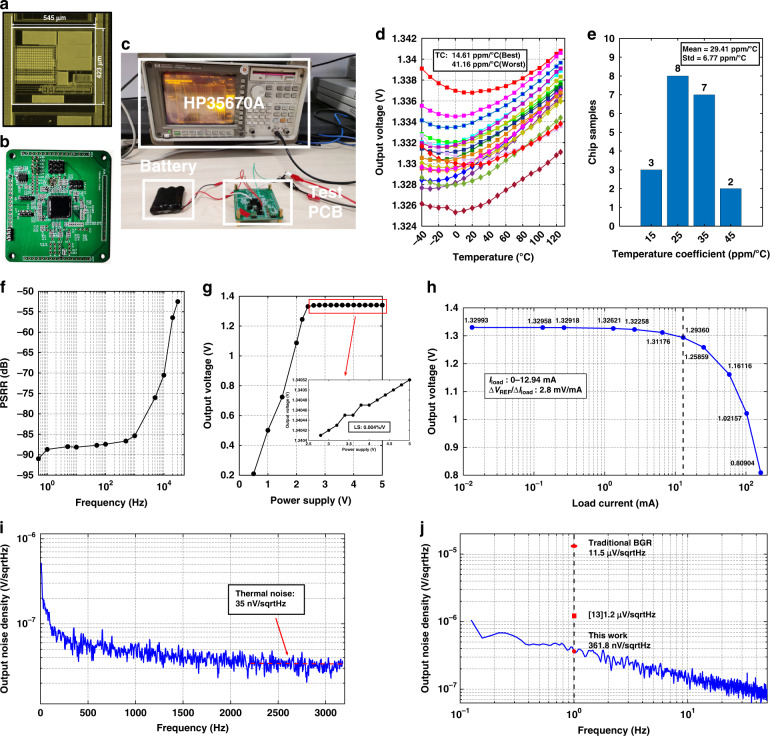
Measurement results of proposed BGR. **a** Chip photo of the proposed BGR. **b** Photo of the test PCB. **c** Noise performance test environment. **d** Temperature behavior of 20 tested chips. **e** Statistical results of the BGR temperature coefficient. **f** PSRR performance of the proposed BGR. **g** Load regulation performance of the proposed BGR. **h** Load regulation performance of the proposed BGR. **i** Noise performance of the proposed BGR. **j** Low-frequency noise performance of the proposed BGR, traditional voltage-mode BGR, and the BGR in ref. ^13^

The area of this BGR is 545 × 423 μm. The chip photo is shown in Fig. 6a, and Fig. 6b presents the test printed circuit board (PCB). Twenty samples from four wafers are utilized to carry out the temperature test. The temperature behaviors of the 20 chips are illustrated in Fig. 6d. Over a range from −40 to 125 °C, the best temperature coefficient (TC) is 14.61 ppm/°C, and the worst TC is 41.16 ppm/°C. Statistical results in Fig. 6e shows that the mean value of TC is 29.41 ppm/°C, and the standard deviation (1*σ*) is 6.77 ppm/°C. Process error is the main reason for TC deterioration. When the process error changes the designed ratio of *R*_*1*_ versus *R*_*3*_, *ΔV*_BE_ cannot completely eliminate the first-order temperature term of *V*_BE_, and the residual first-order temperature term increases the TC.

The PSRR performance of the proposed BGR is tested from 0.5 Hz to 80 kHz, as shown in Fig. 6f. The PSRR is −90.97 dB at 0.5 Hz and −70.52 dB at 10 kHz. Even if there is no error amplifier, the proposed BGR can achieve good PSRR performance due to the negative feedback loop and the PTAT current. The BGR of a high PSRR can repress the noise source coupled with other instances in the MEMS gyroscope system, which is also important for a high-performance MEMS gyroscope. Figure 6g presents the load regulation performance of the proposed BGR. When the supply voltage varies from 2.8 to 5 V, the variation in output voltage is 0.12 mV, and the BGR line sensitivity is 0.004%/V.

Unlike other voltage-mode BGRs, this design can deliver a large load current. Figure 6h illustrates the relationship between the output voltage and load current. When the load resistor decreases to 100 Ω, the output current increases to 12.94 mA. Over the range of load current, the variation of the output voltage is 36.33 mV. However, when the load current continues to increase, the source follower *M*_*1*_ in Fig. 3a cannot sustain the output voltage anymore. Therefore, the proposed BGR can achieve a load regulation of *∆V*_REF_/*∆I*_Load_ = 2.8 mV/mA up to 12.94 mA.

The noise performance test was carried out as shown in Fig. 6c. To reduce the effect of the supply voltage noise on the BGR output voltage, an ultralow noise battery is applied as the power supplier. Using the dynamic signal analyzer (HP35670A), the noise density spectrum of the proposed BGR is as shown in Fig. 6i. The thermal noise density of this BGR is 35 nV/√Hz. To calculate the low-frequency noise accurately, the low-frequency region noise density spectrum of this BGR is illustrated in Fig. 6j. The noise density at 1 Hz is 361.8 nV/√Hz. The work in ref. ^13^ also reduces the amplification factors, and its noise density at 1 Hz is 1.2 μV/√Hz. In addition^13^, it provides the simulation results of traditional voltage-mode BGR noise performance. Its noise density at 1 Hz is 11.5 μV/√Hz. Therefore, the proposed BGR in this paper further reduces the low-frequency noise compared to previous work. The integrated noise of this BGR from 0.1 Hz to 10 Hz is 0.82 μV.

### Bias instability test results

**Fig. 7:**
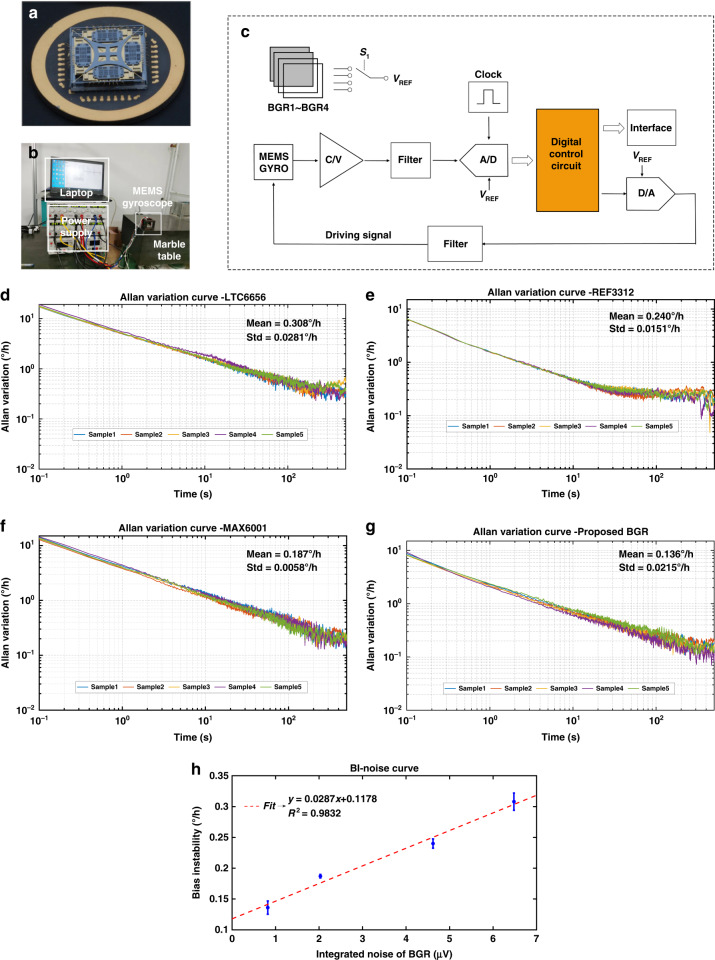
Measurement results of MEMS gyroscope with different BGRs. **a** Die photo of the MEMS gyroscope. **b** Bias instability test environment. **c** Readout circuit of the MEMS gyroscope. **d** Allan variance curve with commercial BGR LTC6656. **e** Allan variance curve with commercial BGR REF3312. **f** Allan variance curve with commercial BGR MAX6001. **g** Allan variance curve with the proposed BGR. **h** The curve of BI versus BGR’s 1/f noise

To verify the effect of the BGR’s 1/f noise on the gyroscope’s BI, the proposed BGR and some commercial BGRs are utilized to carry out bias instability tests with the same MEMS gyroscope^20^. Figure 7a presents the die photo of the MEMS gyroscope. To eliminate the effect of environmental vibration on the gyroscope’s zero-rate output, the MEMS gyroscope is fixed on a hexahedron made of aluminum and placed on a marble table, as shown in Fig. 7b. The readout circuit of the gyroscope is illustrated in Fig. 7c, and different BGR swaps in through the switch circuit to provide a reference voltage.

At room temperature, the MEMS gyroscope’s output angular rate is recorded for 1 h at a sample rate of 10 Hz. The Allan variance is calculated from the sample data. The Allan variance curves with each BGR are illustrated in Fig. 7d–g. When the BGR’s low-frequency integrated noise (0.1 to 10 Hz) decreases from 6.37 to 0.82 μV, the bias instability of the MEMS gyroscope improves from 0.308°/h to 0.136°/h. The relationship between the BGR’s 1/f noise and the gyroscope’s BI is presented in Fig. 7h, which demonstrates that reducing the BGR’s 1/f noise can nearly linearly improve the gyroscope’s BI.

## Discussion

The performance parameters of the proposed BGR and previously reported work are listed in Table 1. Obviously, by removing the error amplifier and optimizing the device parameters, the proposed BGR achieves a lower 1/*f* noise, which makes the proposed BGR suitable for high-precision applications, especially high-performance MEMS gyroscopes. However, the limitation of this BGR is a comparatively large TC since the BGR has no trimming circuits and high-order temperature compensation circuits. Lowering the TC needs to be studied in the future.

**Table 1:** Performance comparison of BGRs

	This work	^12^ JSSC 2021	^13^ TCAS-I 2019	^21^ JSSC 2019	^22^ JSSC 2018	^23^ TED 2015	^9^ JSSC 2011
Process	**180** **nm CMOS process**	180 nm CMOS process	350 nm CMOS process	180 nm CMOS process	350 nm CMOS process	180 nm CMOS process	160 nm CMOS process
Area (mm^2^)	**0.23**	0.38	0.0616	0.0045	0.23	0.05	0.12
Supply voltage (V)	**2.8-5**	2–3.3	2.6–5	1.0	3.3	1.3–2.6	1.8
Output voltage (V)	**1.3315**	1.1419	2.47	0.6926	1.22	1.1402	1.0875
Line sensitivity (%/V)	**0.004**	NA	0.0041	0.02	NA	0.03	NA
PSRR (dB@Hz)	**-90.97** dB **@0.5** Hz, **-70.52** dB **@10** kHz	−76 dB @DC	−83 dB @DC	−55 dB @100 Hz	NA	−54 dB @100 Hz	−74 dB @DC
Current consumption (mA)	**0.1787**	0.017	0.094	NA (192 pW)	0.115	0.0043	0.055
Best temperature Coefficient (ppm/°C)	**14.61 (-40–125** **°C)**	4.3 (-40–150 °C)	0.9 (-45–125 °C)	33 (average) (-20–100 °C)	NA	4.1 (−55–125 °C)	5 (−40–125 °C)
Trimming	**No**	No	Yes	No	Yes	Yes	Yes
0.1–10 Hz Integrated Noise (V)	**0.82μ**	56 μ	3.6 μ	26.8 μ	7.84 μ	10.23 μ	6.1 μ
Output Current (mA)	**12.94**	NA	0.03	NA	NA	20	NA

To verify the effect of BGR’s 1/f noise on the MEMS gyroscope’s BI, bias stability tests with the proposed BGR and commercial BGRs were carried out. The parameters of these BGRs are listed in Table 2. To avoid the influence of the BGR’s temperature drift on the gyroscope’s output, the proposed BGR and the chosen commercial BGR have approximate TC but different 1/*f* noise. The experimental setup is that different BGR swaps in through a switch circuit, and other parts in the circuit are the same. Furthermore, we repeat the bias stability tests to avoid the influence of the experimental setup and environmental interference. Statistical results demonstrate that when BGR’s integrated noise decreases from 6.48 to 0.82 μV, the gyroscope’s BI improves from 0.308°/h to 0.136°/h. Reducing the BGR’s 1/f noise can nearly linearly improve the gyroscope’s BI. Of course, the gyroscope’s BI cannot be reduced infinitely since it is also affected by other instances’ 1/f noise, such as the ADC. We will study to further improve the gyroscope’s BI in other aspects.

**Table 2:** Parameters of the proposed BGR and some commercial BGRs

	Integrated output noise (0.1–10 Hz)	Temperature coefficient (-40–125 °C)	Mean value of BI	Standard variation value of BI
LTC6656	6.48 uV	19.28 ppm/°C	0.308°/h	0.0281°/h
REF3312	4.62 uV	18.92 ppm/°C	0.240°/h	0.0151°/h
MAX6001	2.03 uV	18.47 ppm/°C	0.187°/h	0.0058°/h
The proposed BGR	0.82 uV	19.91 ppm/°C	0.136°/h	0.0215°/h

## Materials and methods

In this paper, the parameters of this BGR are given as follows: *R*_*1*_ = 5 kΩ, *R*_*2*_ = 20 kΩ, *R*_*3*_ = 1.5 kΩ, *R*_*9*_ = *R*_*10*_ = *R*_*4*_ = *R*_*5*_ = 20 kΩ, *R*_*6*_ = 2.5 kΩ, *R*_*7*_ = 500 kΩ, *M*_*1*_ (L = 600 nm, W = 10 mm, finger = 16, multiplier = 32), *M*_*1*_ (L = 500 nm, W = 10 mm, finger = 8, multiplier = 1), *n* = 64. The proposed BGR has been implemented in a 180 nm CMOS process. The specific structure of the MEMS gyroscope is shown in ref. ^20^, and the MEMS gyroscope has been fabricated in a silicon-on-glass (SOG) process. The readout circuit illustrated in Fig. 7c is implemented by commercial instances. ADA4177, as a high-precision amplifier, is applied to achieve the C/V circuit and filters. AD7690 as the high-precision ADC and DAC8812 as the high-precision DAC are used.
